# Tissue-Specific Analysis of Glycogen Synthase Kinase-3α (GSK-3α) in Glucose Metabolism: Effect of Strain Variation

**DOI:** 10.1371/journal.pone.0015845

**Published:** 2011-01-06

**Authors:** Satish Patel, Katrina Macaulay, James R. Woodgett

**Affiliations:** 1 Samuel Lunenfeld Research Institute, Mount Sinai Hospital, Toronto, Ontario, Canada; 2 Department of Medical Biophysics, University of Toronto, Toronto, Ontario, Canada; University of Las Palmas de Gran Canaria, Spain

## Abstract

**Background:**

Over-activity and elevated expression of glycogen synthase kinase-3 (GSK-3) has been implicated in the etiology of insulin resistance and Type 2 diabetes. Administration of specific GSK-3 inhibitors to diabetic or obese rodent models improves glycaemic control and insulin sensitivity. However, due to the indiscriminatory nature of these inhibitors, the relative contribution of the two isoforms of GSK-3 (GSK-3α and GSK-3β) is not known. Recently, we demonstrated that an out-bred strain of mice (ICR) lacking expression of GSK-3α in all tissues displayed improved insulin sensitivity and enhanced hepatic glucose metabolism. We also found that muscle (but not liver) inactivation of GSK-3β conferred insulin and glucose sensitization in an in-bred strain of mice (C57BL/6).

**Methodology/Principal Findings:**

Here, we have employed tissue-specific deletion of GSK-3α, to examine the relative contribution of two insulin-sensitive tissues, muscle and liver, towards the insulin sensitization phenotype originally observed in the global GSK-3α KO animals. We found that mice in which GSK-3α has been inactivated in either skeletal-muscle or liver displayed no differences in glucose tolerance or insulin sensitivity compared to wild type littermates. Given the strain differences in our original analyses, we examined the insulin and glucose sensitivity of global GSK-3α KO animals bred onto a C57BL/6 background. These animals also revealed no significant differences in glucose metabolism/insulin sensitivity compared to their wild type littermates. Furthermore, deletion of hepatic GSK-3α on the out-bred, ICR background failed to reproduce the insulin sensitivity manifested by the global deletion of this isoform.

**Conclusions/Significance:**

From these data we conclude that the improved insulin sensitivity and hepatic glucose homeostasis phenotype observed upon global inactivation of GSK-3α is strain-specific. We surmise that the insulin-sensitization observed in the out-bred strain of mice lacking GSK-3α is mediated by indirect means that do not require intrinsic function of GSK-3α in skeletal muscle and liver tissues.

## Introduction

Glycogen synthase kinase-3 (GSK-3) is a ubiquitously expressed serine/threonine protein kinase that is encoded by two distinct genes, GSK-3α (52 kDa) and GSK-3β (47 kDa). These two isoforms are highly conserved and share ∼98% sequence similarity in their catalytic domains [1]. GSK-3 is a constitutively active kinase in resting cells that becomes rapidly inactivated by phosphorylation at Ser 21 (GSK-3α) and Ser 9 (GSK-3β) in response to insulin through a phosphatidylinositol 3 (PI-3) kinase/protein kinase B (PKB, also termed Akt)-dependent manner.

Both GSK-3 expression and activity are elevated in muscle and adipose tissue of diabetic humans and rodents [2], [3]. In addition, GSK-3 inhibitors improve insulin sensitivity in rodent models of diabetes, alleviating hyperglycaemia by decreasing hepatic gluconeogenesis and stimulating glycogen synthesis [4], [5], [6]. Furthermore, novel peptide inhibitors of GSK-3 (L803-mts) reverse the diabetic state associated with the *ob/ob* mouse model [7]. Interestingly, generation of mice expressing insulin-insensitive mutants of GSK-3 (conversion of Ser 21 of GSK-3α and Ser 9 of GSK-3β to alanine; S21A/S9A), does not result in a diabetic phenotype [8].

While the two GSK-3 isoforms are structurally similar, they are not functionally equivalent. Mice lacking GSK-3β expression die during embryogenesis (E13.5-16.5) displaying severe liver apoptosis and heart patterning defects [9], [10]. Conversely, GSK-3α knockout (KO) animals are viable and fertile and exhibit enhanced insulin sensitivity and glucose tolerance, accompanied by elevated hepatic glycogen deposition [11]. Interestingly, although insulin-stimulated PKB and GSK-3β phosphorylation was significantly increased in livers of GSK-3α KO animals, muscle insulin signaling was unaffected by the loss of GSK-3α. By contrast, skeletal muscle-specific inactivation of GSK-3β resulted in improved glucose tolerance, enhanced muscle glycogen deposition and insulin-stimulated GS activity [12]. However, mice harbouring liver-specific inactivation of GSK-3β exhibited normal metabolic characteristics [12]. Pancreatic deletion of the same isoform alleviated hyperglycaemia in IRS-2 KO mice [13]. The data from these studies support the idea that there are isoform and tissue-specific roles for GSK-3 in the regulation of glucose metabolism and insulin action, such that GSK-3α is the predominant regulator of hepatic GS and glycogen synthesis while GSK-3β has more prevalent role in these processes within skeletal muscle and pancreatic islet tissue. However, it remains unclear as to whether the anti-diabetic phenotype observed in GSK-3α KO animals is a direct effect of GSK-3α loss in insulin-sensitive tissues, such as the liver, or whether the insulin sensitization is a consequence of the functional loss of GSK-3α in other tissues. To address this question, we have engineered the conditional mouse line from which the global GSK-3α KO was developed, and here, describe the generation of skeletal muscle- and liver-specific GSK-3α KO mouse models. Our analysis spans several strains of mouse commonly used in studies of insulin sensitivity. The original strain employed for the global GSK-3α knockout [11] was an out-bred strain termed ICR. The tissue-specific knockouts of GSK-3β were initially reported [12] on the C57Black/6 in-bred strain (hereafter termed C57BL/6). In addition, tissue-specific Cre animals were on mixed C57BL/6 and 129 (both in-bred) background. Here, we report that on the C57BL/6 strain, skeletal muscle deletion of GSK-3α does not result in increased insulin sensitivity. Similarly and unexpectedly, the liver-specific GSK-3α KO also lacks obvious sensitization towards insulin or glucose. These observations led us to examine whether there are strain-specific effects associated with the ability of GSK-3α to regulate glucose metabolism. We conclude that the anti-diabetic phenotype observed in the global GSK-3α KO animals is not a result of a direct effect of inactivation of GSK-3α in either the muscle or liver and is strain-dependent.

## Materials and Methods

### Ethics Statement

The animal experimentation conducted during this study was performed under the guidance and approval of the Toronto Centre for Phenogenomics (TCP) animal care committee under protocol #0047a-H. This protocol minimized any suffering for the mice used through use of analgesics where appropriate. All procedures were subject to regular external evaluation by the Canadian Council for Animal Care.

### Mouse strains

The GSK-3α floxed and the GSK-3α global knockout mice were generated as previously described [11]. The global deletions of exon 2 of GSK-3α were bred onto both the ICR and C57BL/6 backgrounds whereas the GSK-3α floxed animals were generated on a C57BL/6 background only (most breedings exceeded 6 back-crosses to ensure strain purity and employed heterozygous animals and genotyping). Skeletal-muscle specific GSK-3α knockout animals were generated by breeding the conditionally targeted GSK-3α floxed mice with mice expressing Cre under the control of the myosin light chain 1*f* (MLC1*f*) promoter (kindly provided by Steve Burden, Skirball Institute, NY as previously described [12], [14]. These GSK-3α flx/flx MLC1*f*-cre + mice (C57BL/6/129 background: F5 Backcross) are denoted MLC Cre +. To generate liver-specific GSK-3α knockout animals, the GSK-3α floxed mice were crossed to the C57BL/6 Cg-Tg(Alb-Cre)21Mgn/J (JAX lab) strain which carries Cre recombinase under the control of the albumin promoter as previously described [12], [15]. These GSK-3α flx/flx AlbCre + mice (C57BL/6/129 background: F5 backcross) are designated Alb Cre +. Genotypes for the various tissue-specific deletions were confirmed by PCR for the presence of Cre and for the detection of the deleted, floxed GSK-3α allele. The GSK-3α flx/flx lines demonstrated similar expression of GSK-3 protein as well as other components of the insulin-signaling pathway when compared to wild type (WT) animals (data not shown). We performed additional experiments to demonstrate that the presence of the Alb-Cre or MLC-Cre transgene in animals wild type with respect to GSK-3 had no significant effects on the metabolic profiles (as assessed by glucose tolerance (GTT) and insulin tolerance tests (ITT)) analyzed in these studies (data not shown). In addition, all studies were performed with respective littermate controls to control for possible environmental influences.

Male animals were housed 5 per cage under a light/dark cycle of 12 hours each, in the Toronto Centre for Phenogenomics (TCP) animal facility, with free access to food and water except where noted. Individual body weights were monitored over a 22-week period. For confirmation of genotypes, genomic DNA prepared from tail snips was analyzed by PCR.

### Glucose and Insulin Tolerance Test

Mice were fasted overnight (16–18 h) and administered 2 mg/g glucose by intraperitoneal injection (i.p.) for glucose tolerance testing and fasted for 5 h prior to i.p. injection of 1 mU/g insulin for insulin tolerance testing. Blood glucose was assayed from the tail vein using the OneTouch UltraSmart blood glucose monitoring system (Lifescan, Canada) at the indicated times.

### Plasma Insulin ELISA

Blood was collected from the tail vein of random-fed mice, or following an overnight (16–18 h) fast. Plasma was assayed for insulin using a rat/mouse insulin enzyme-linked immunosorbent assay kit (ALPCO diagnostics).

### Preparation of Tissue Lysates

Mice were fasted overnight and either treated with 150 mU/g insulin or 2 mg/g glucose for 15 min by i.p. injection. Following experimental manipulation, tissues were extracted and lysed in lysis buffer (150 mM NaCl, 1% NP-40, 0.5% DOC, 0.1% SDS, 1 mM EDTA, 50 mM Tris, pH 8.0, 10 mM NaF, 1 mM Na-orthovanadate, 10 mM β-glycerophosphate and one ‘Complete’ protease inhibitor cocktail tablet/50 ml) using a 2 ml Kontes™ Tissue grinder. Lysates were centrifuged (16,000 *g*, 4°C for 15 min) and protein concentration determined by Biorad DC protein assay.

### Immunoblotting

Tissue lysates (20 µg) were subjected to SDS-PAGE and proteins transferred onto PVDF membrane. Membranes were blocked for 1 h at room temperature in 5% non-fat milk/Tris-buffered saline (50 mM Tris-HCl, 150 mM NaCl, pH 7.4) containing 0.1% Tween-20 prior to probing with antibodies directed against the following antigens: GSK-3α/β (1∶10,000, Biosource), phospho-GSK-3β (1∶1000, Cell Signaling), β-catenin (1∶1000, BD Transduction Laboratories), PKB (1∶1000, Cell Signaling), phospho-PKB Ser^473^ (1∶1000, Cell Signaling Technologies), GS (GYS1, 1∶1000, Chemicon), GS (GYS2, 1∶1000, gift from J. Guinovart, University of Barcelona), phospho-GS (1∶1000, Cell Signaling Technologies), β-actin (1∶20,000, Abcam), GAPDH (1∶100,000, Abcam), overnight at 4°C. Membranes were washed 3 times in Tris-buffered saline/0.1% Tween-20 prior to incubation with HRP-anti rabbit IgG (1∶10,000) or HRP-anti mouse IgG (1∶10,000) antibodies. Visualization was performed using enhanced chemiluminescence reagent exposure to Kodak autoradiographic film. Densitometry quantification was carried out using an Alpha Innotech FluorChem HD2.

### Glycogen Synthase Activity Assay

Frozen skeletal muscle and liver tissue was minced using 1 ml homogenization buffer (50 mM Tris pH 7.8, 100 mM NaF, 10 mM EDTA, 5% glycerol and protease inhibitor cocktail tablet) and homogenized using a Brinkman Polytron. The tissue homogenates were cleared by centrifugation at 3,000 *g* for 10 min at 4°C and glycogen synthase activity was assayed from the supernatants according to the method of Thomas *et al*. [16] based on the incorporation of uridine 5-diphosphate (^3^H) glucose (UDPG) into glycogen.

### Glycogen Content

25 mg of muscle or liver tissue was acid hydrolyzed in 2 N HCl by heating it at 95°C for 2 h and neutralized using 2 N NaOH. The liberated free-glycosyl units were assayed spectrophotometrically by using a glucose reagent hexokinase-dependent-assay kit (Amresco, OH) as previously described (4). Glycogen was also visualized in paraffin-embedded tissue sections by using a periodic acid-Schiff (PAS) staining kit (Sigma Aldrich). Tissue sections were also stained with standard hematoxylin and eosin for general cell morphology.

### GSK-3 Activity Assays

Assays of GSK-3 were performed on mouse tissues extracts as described previously [12]. Briefly, 100 mg of muscle or liver tissue was lysed in 3 ml of detergent-free lysis buffer (50 mM Tris-HCl, 4 mM EDTA, 2 mM EGTA, 10 mM Naβ-glycerophosphate, 5 mM NaF, 1 mM PMSF, 1 mM Na-orthovanadate, protease and phosphatase inhibitor cocktails, pH 7) and homogenised using a 2 ml Kontes™ Tissue grinder. The tissue homogenate were centrifuged at 4,000 g for 30 min at 4°C and the supernatant applied to a CM-Sepharose fast flow resin column (Amersham) pre-equilibrated with 25 mM Tris-HCl, 2 mM EDTA, 1 mM EGTA, 10 mM Naβ-glycerophosphate, 2.5% glycerol, 5 mM NaF, 1 mM PMSF, 1 mM orthovanadate, protease and phosphatase inhibitors cocktail, pH 7 (buffer A). The column was washed with 5 column volumes of low-salt wash buffer (buffer A +20 mM NaCl) and GSK-3 eluted using buffer A +250 mM NaCl. Fractions containing the highest concentrations of protein were pooled and used in a kinase assay to assess GSK-3 activity as described in [17].

### Statistical Analysis

For multiple comparisons statistical analysis was performed using either two way analysis of variance (ANOVA) followed by a Bonferroni post test or unpaired student's t-tests. Data analysis was performed using GraphPad Prism software and considered statistically significant at P values <0.05, unless otherwise stated.

## Results

**Figure 1:**
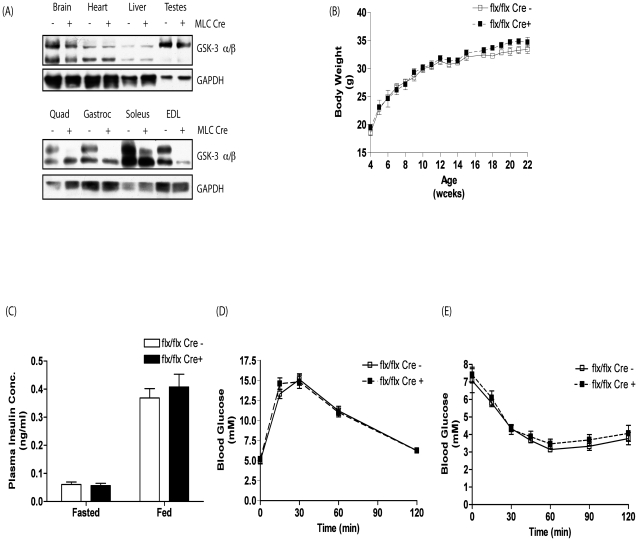
GSK-3 expression, body weight, plasma insulin concentration, glucose tolerance and insulin sensitivity in C57BL/6/129 GSK-3α muscle KO animals. (A) Brain, heart, liver, testes and quad, gastroc, soleus and EDL muscles and (B) brain, heart, testes, liver and gastroc from eight week old MLC Cre − (−) and MLC Cre + (+) littermate control animals were extracted and lysed as described in Experimental Procedures and proteins resolved by SDS-PAGE. Proteins were detected by immunoblotting with antibodies against GSK-3α/β. Even loading was determined using antibodies to GAPDH. Blots are representative from three separate experiments. (B) Body weight of male MLC Cre − (open squares) and MLC Cre + (filled squares) littermate control mice was monitored weekly from age 4 to 22 weeks. Values are the mean ± SEM from eight separate animals. (C) Plasma insulin concentration was determined in eight week old male MLC Cre − (open bars) and MLC Cre + (filled bars) littermate control animals under fasted and fed conditions. Values are the mean ± SEM from at least seven separate animals. Blood glucose concentration in eight week old male MLC Cre − (open squares) and MLC Cre + (filled squares) littermate control mice was measured at the indicated times following administration of (D) 2 mg/g glucose or (E) 1 mU/g insulin by i.p. injection as described in Experimental Procedures. Values are the mean ± SEM from at least seven separate animals.

Conditional GSK-3α mice that express a modified allele of GSK-3α, where exon 2 is flanked by LoxP sequences, have been previously described [11]. Skeletal muscle-specific GSK-3α knockout (KO) animals were generated by breeding GSK-3α floxed mice (flx/flx –C57BL/6/129 background) with mice expressing Cre under the control of the myosin light chain 1*f* (MLC1*f*) promoter (MLC Cre- C57BL/6B6/129 background), a gene that is predominantly expressed in fast twitch skeletal muscle fibres. The resultant skeletal muscle GSK-3α KO mice (MLC Cre +) are viable, fertile and born to the expected Mendelian frequency. As expected, GSK-3α expression is completely lost in quadricep (quad), gastrocnemius (gastroc) and extensor digitorum longus (EDL) muscles, whereas there is only partial reduction of GSK-3α expression in soleus, which is primarily composed of slow twitch fibres (Figure 1A). Importantly in the GSK-3α flx/flx MLC Cre + (MLC Cre +) mice, loss of GSK-3α expression is restricted to skeletal muscle tissue only and remains unaltered in brain, heart, liver and testes of mice when compared to GSK-3α flx/flx MLC Cre - (MLC Cre -) (Figure 1A). Weekly weight analysis from age 4–22 weeks revealed no significant differences in whole body weight between MLC Cre + and MLC Cre - littermate control animals (Figure 1B).

To determine whether muscle-specific loss of GSK-3α expression alters whole body metabolism, the concentration of plasma insulin, glucose tolerance and insulin sensitivity were determined. Fasted and random fed plasma insulin concentrations were comparable between MLC Cre + and MLC Cre - littermate control animals (Figure 1C). Furthermore, both glucose tolerance and insulin sensitivity showed no significant differences in 8 week (or 6 month (data not shown)) old male MLC Cre + and MLC Cre - littermate control mice (Figures 1D and 1E, respectively) demonstrating that the muscle-specific inactivation of GSK-3α has minimal effects on glucose tolerance and insulin sensitivity.

**Figure 2:**
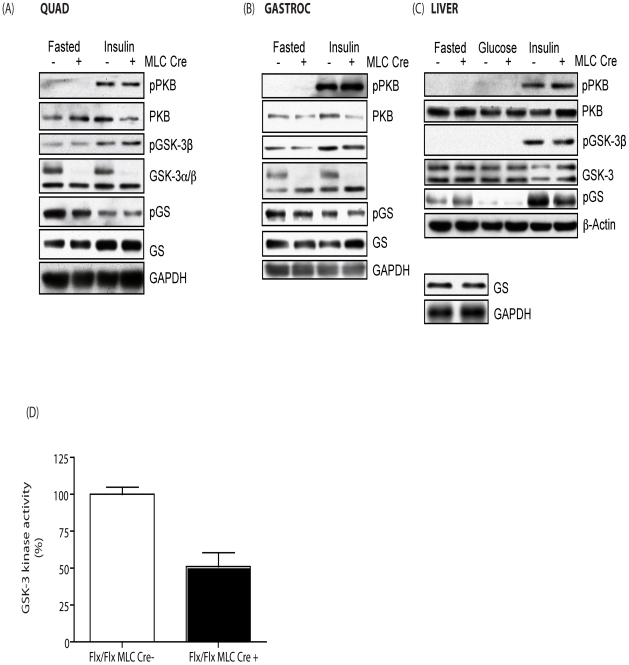
Effect of GSK-3α muscle KO on insulin signaling. (A) Quad, (B) gastroc and (C) liver from eight week old MLC Cre − (−) and MLC Cre + (+) littermate control animals was extracted following either an over night (16–18 h) fast alone or an overnight fast followed by i.p. administration of 150 mU/g insulin for 15 min. Twenty µg of the protein lysate was resolved by SDS-PAGE. Proteins were detected by immunoblotting with antibodies against phospho-PKB (pSer473), PKB, phospho-GSK-3β (pSer9), GSK-3β, phospho-GS (pSer641) and GS (lower left inset in C). Loading was determined using antibodies to either GAPDH or β-actin. Blots are representative from five separate experiments. (D) GSK-3 kinase activity was determined from muscle tissue extracted from male MLC Cre − control (open bars) and MLC Cre + (filled bars) mice as described in experimental procedures. GSK-3 kinase activity was determined using a quantitative peptide phosphorylation assay. GSK-3 kinase activity is expressed relative to the MLC Cre- control (which is set at 100%) and is the mean ± SEM of four different muscle samples with each assayed in triplicate. Genetic background was C57BL/6/129.

Since GSK-3 is a key component of the insulin-signaling pathway, we assessed whether muscle-specific loss of GSK-3α expression alters insulin signaling. Insulin administration results in a significant increase in PKB/Akt and GSK-3β phosphorylation. Muscle-specific loss of GSK-3α did not alter fasted or insulin-stimulated PKB or GSK-3β phosphorylation status or total expression of either protein in skeletal muscle tissues (Figures 2A and 2B, respectively). Assay of GSK-3 activity from MLC Cre + skeletal muscle extracts revealed a 50% reduction in kinase activity compared to MLC Cre – control animals (Figure 2C).

**Figure 3:**
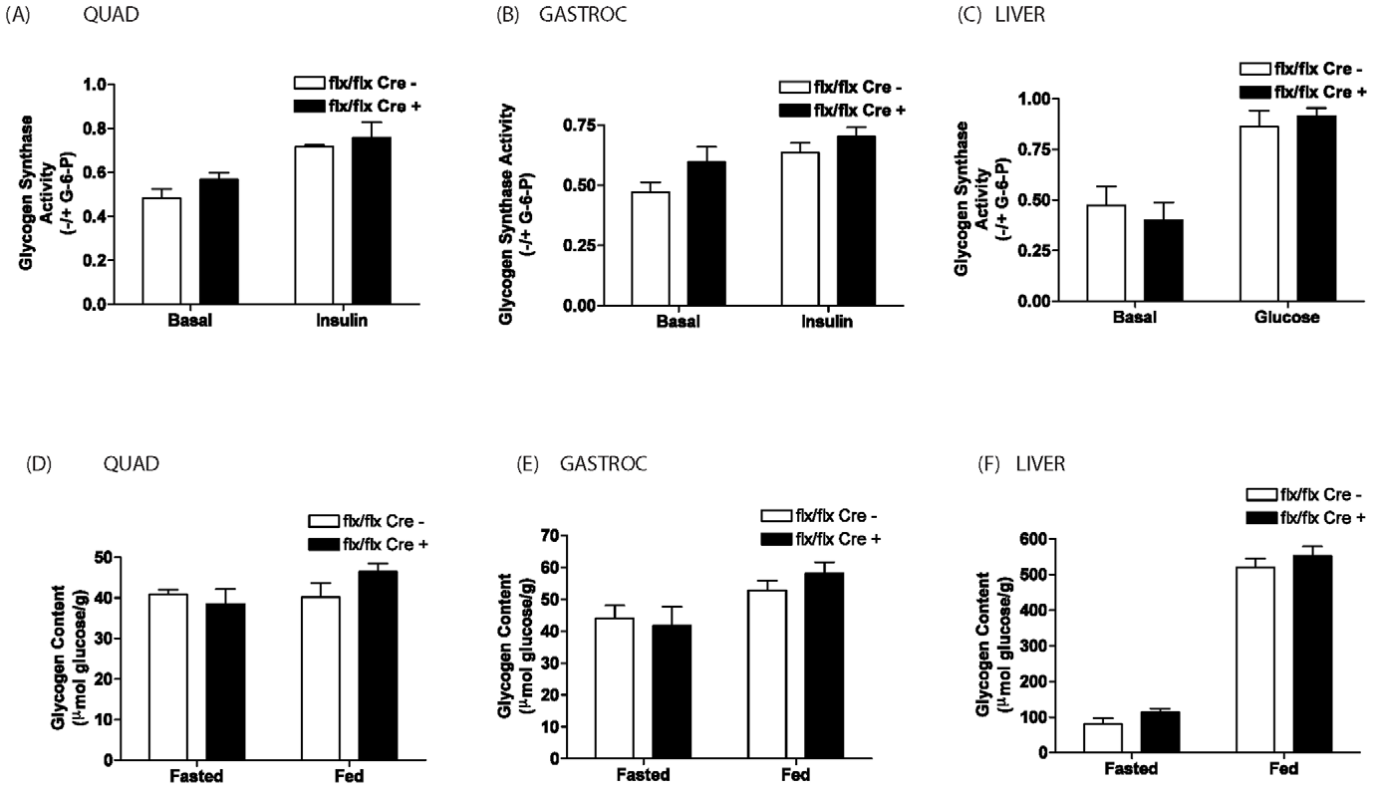
Glycogen synthase activity in quad, gastroc and liver of GSK-3α muscle KO animals. Glycogen synthase activity was determined in (A) quad, (B) gastroc and (C) liver of eight weeks old MLC Cre – (open bars) and MLC Cre + (filled bars) tissues by assaying incorporation of glucose from uridine diphospho-{6-^3^H)-D-glucose into glycogen and expressed as a ratio of activity in the absence divided by that in the presence of glucose-6-phosphate. Values are the mean ± SEM for at least five experiments carried out in duplicate. Glycogen content was measured in (D) quad, (E) gastroc and (F) liver from eight week old MLC Cre − (open bars) and MLC Cre + (filled bars) following either an overnight fast or random feeding. Tissues were extracted, acid-hydrolyzed and glycosyl units assayed using a glucose reagent hexokinase method (Amresco, Ohio) as described in Experimental Procedures. Glycogen content is expressed as µmol glucose/g tissue. Values are mean ± SEM from at least five separate animals with each assayed in triplicate. Genetic background of the animals was C57BL/6/129.

GS is dephosphorylated and activated in response to an insulin signal in muscle and to a glucose-signal in the liver [18]. However, fasted and insulin- or glucose-induced GS dephosphorylation in muscle or liver respectively is comparable between MLC Cre + and MLC Cre - tissues (Figure 2). Furthermore, in line with the phosphorylation status, fasted and insulin-stimulated GS activity is similar in quad and gastroc tissues of MLC Cre + compared to MLC Cre - animals (Figures 3A and 3B). As expected, fasted and glucose-induced activation of hepatic GS is also comparable between MCL Cre + and MLC Cre – mice (Figure 3C). In agreement with these data, we found no significant difference in the amount glycogen deposited in muscle or liver under fasted or fed conditions (Figures 3D–F). Together, these data support the conclusion that the selective inactivation of GSK-3α in skeletal muscle tissue has little impact on whole body glucose tolerance and insulin sensitivity.

**Figure 4:**
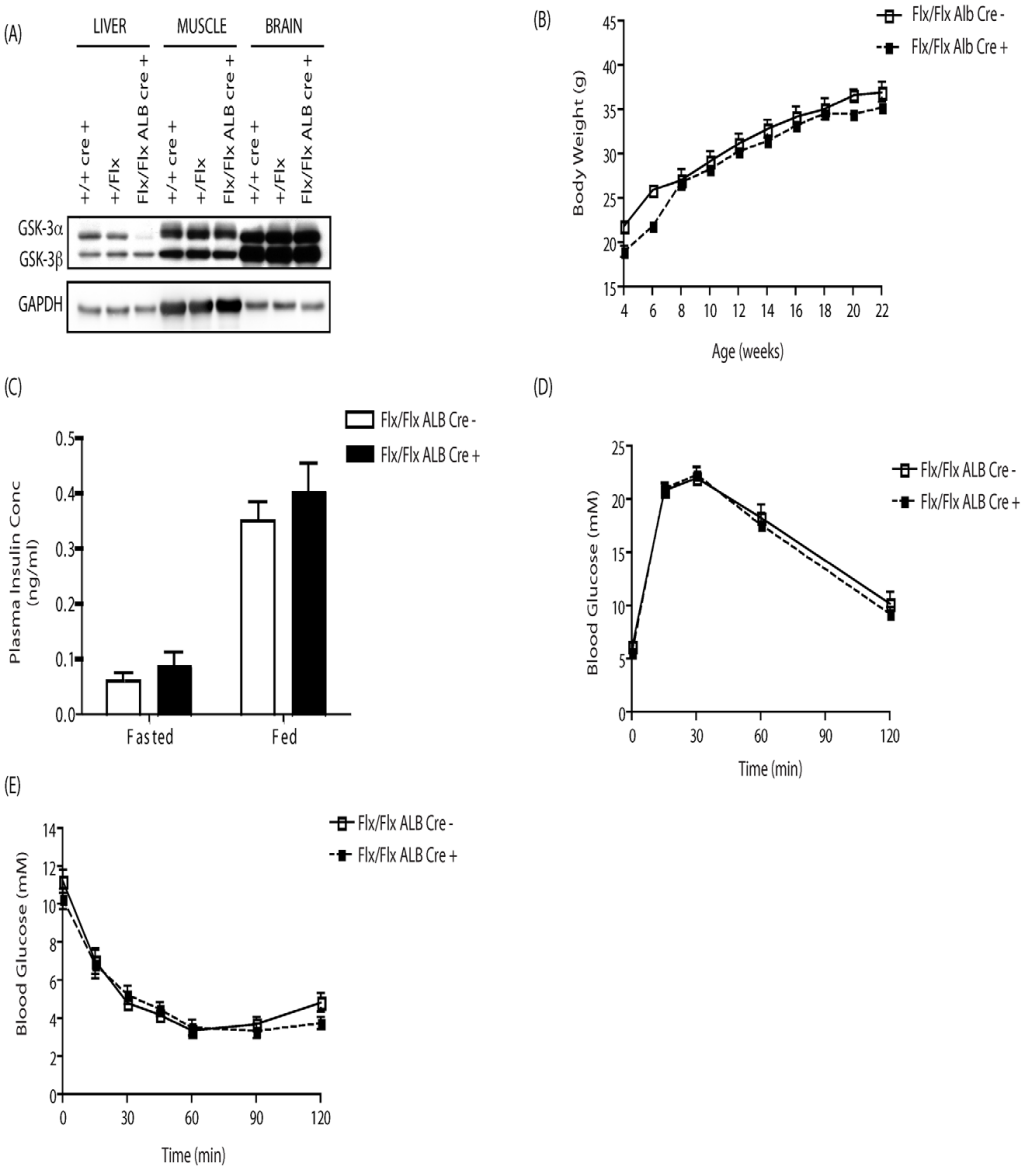
GSK-3 expression, body weight, plasma insulin concentration, glucose tolerance and insulin sensitivity in GSK-3α liver KO animals. Tissue extracts were prepared from eight wk old male mice of the genotypes indicated and immunoblotted with antibodies against (A) GSK-3α/β. Even loading was determined using antibodies to GAPDH. Blots are representative from three separate experiments. (B) Body weight of male Alb Cre − (open squares) and Alb Cre + (filled squares) littermate control mice was monitored weekly from age 4 to 22 weeks. Values are the mean ± SEM from nine separate animals. (C) Plasma insulin concentration was determined in eight week old male Alb Cre − (open bars) and Alb Cre + (filled bars) littermate control animals under fasted and fed conditions. Values are the mean ± SEM from at least seven separate animals. Blood glucose concentration in eight week old male Alb Cre − (open squares) and Alb Cre + (filled squares) littermate control mice was measured at the indicated times following administration of (D) 2 mg/g glucose or (E) 1 mU/g insulin by i.p. injection as described in Experimental Procedures. Values are the mean ± SEM from fourteen separate animals for Alb Cre – and nine separate animals for Alb Cre +. Genetic background was C57BL/6/129.

We previously reported that mice that lack GSK-3α in all tissues (global KO) display improved whole body glucose and insulin tolerance and enhanced hepatic glycogen storage and insulin signaling [11]. To determine whether this effect is manifested by the action of GSK-3α in the liver, hepatocyte-specific GSK-3α KO animals were generated by crossing the GSK-3α floxed (C57BL/6/129 background) mice to a transgenic strain expressing Cre under the control of the albumin promoter (C57BL/6 background). Like the global GSK-3 KO mice, the hepatocyte-specific deleted mice (Alb Cre +) are viable, fertile and born to expected frequency. In the Alb Cre + mice, GSK-3α expression is selectively lost in liver but remains equivalent in other tissues when compared to the Alb Cre- control animals (Figure 4A). Furthermore, Alb Cre + mice display similar body weights compared to their Alb Cre – counterparts (Figure 4B).

**Figure 5:**
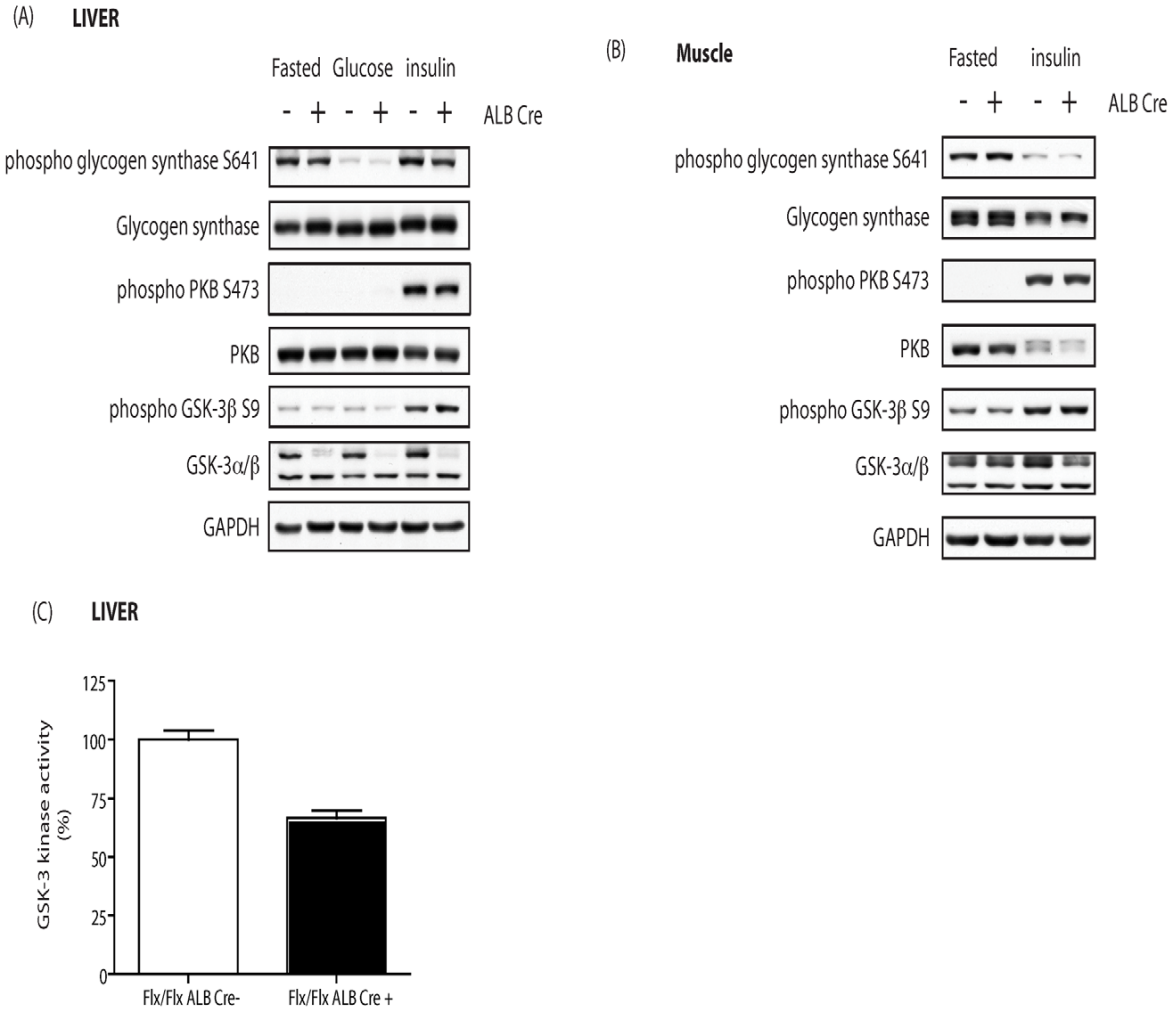
Effect of GSK-3α liver KO on insulin signaling. (A) liver, (B) muscle from eight week old Alb Cre − (−) and Alb Cre + (+) littermate control animals were extracted following either an overnight (16–18 h) fast alone or an overnight fast followed by i.p. administration of 150 mU/g insulin for 15 min. Twenty µg of the protein lysates was resolved by SDS-PAGE. Proteins were detected by immunoblotting with antibodies against phospho-PKB, PKB, phospho-GSK-3β, GSK-3β, phospho-GS and GS. Even loading was determined by blotting for either β-actin or GAPDH. Blots are representative from 4 separate experiments. (C) GSK-3 kinase activity was determined from liver tissue extracted from male Alb Cre − control (open bars) and Alb Cre + (filled bars) mice as described in experimental procedures. GSK-3 kinase activity was determined using a quantitative peptide phosphorylation assay. GSK-3 kinase activity is expressed relative to the Cre− control (which is set at 100%) and is the mean ± SEM of four different liver samples with each assayed in triplicate. Genetic background was C57BL/6/129.

**Figure 6:**
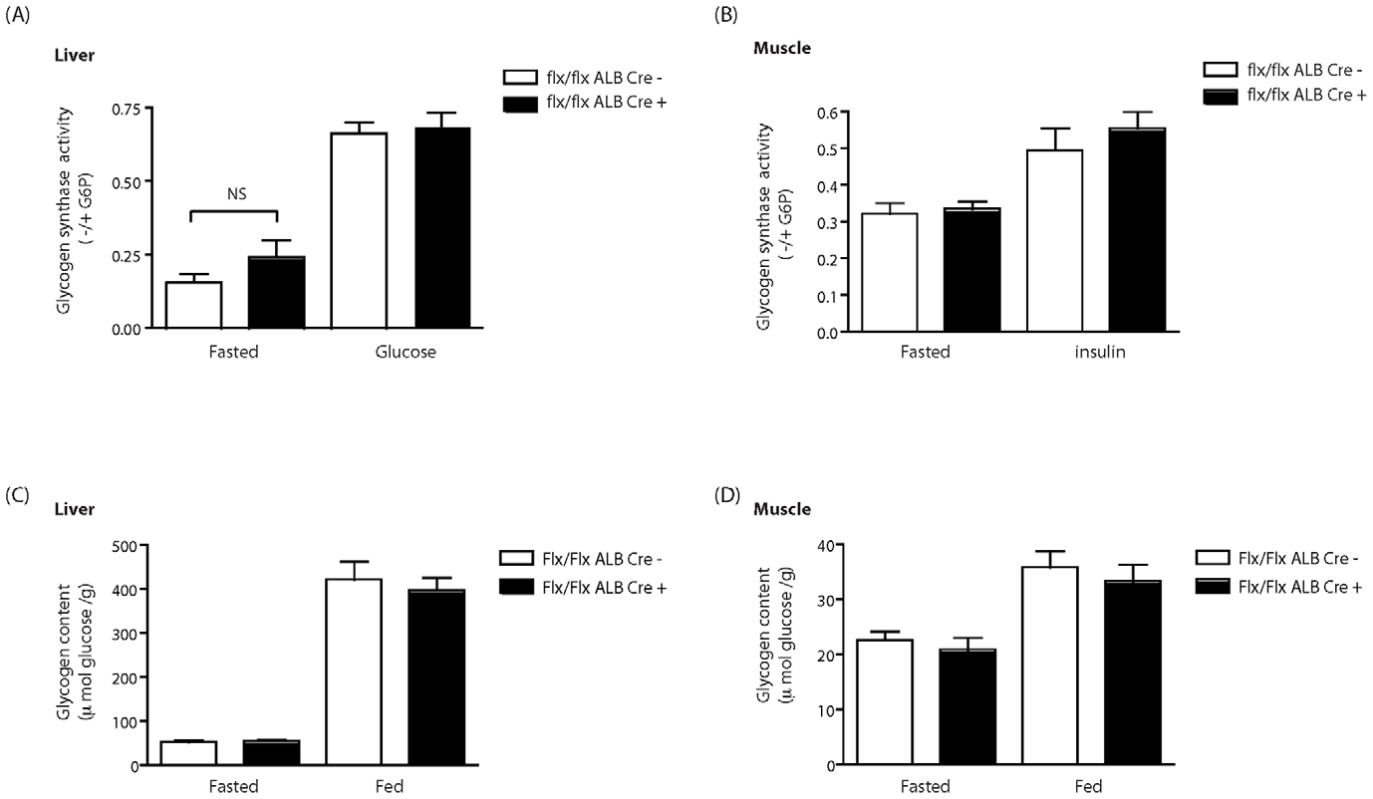
Glycogen synthase activity and glycogen content in muscle and liver of GSK-3 liver KO animals. Glycogen synthase activity was determined in (A) liver and (B) quad of eight weeks old Alb Cre – (open bars) and Alb Cre + (filled bars) tissues by assaying incorporation of glucose from uridine diphospho-(6-^3^H)-D-glucose into glycogen and expressed as a ratio of activity in the absence divided by that in the presence of G-6-P. Values are the mean ± SEM for four separate experiments with each assayed in duplicate. Glycogen content was measured in liver (C) and quad (D) liver from eight week old ALB Cre − (open bars) and ALB Cre + (filled bars) following either an overnight fast or random feeding. Values are mean ± SEM from eight separate animals with each assayed in triplicate. For these experiments, the mouse background strain was C57BL/6/129.

The metabolic phenotype of the hepatocyte-specific GSKα KO was analysed. Measurement of plasma insulin concentration under fasted and random fed conditions revealed no significant differences between 8 week old male Alb Cre + and Alb Cre- control mice (Figure 4C). Interestingly, unlike the global GSK-3α KO, hepatocyte-specific deletion of GSK-3α revealed no statistically significant differences in the rate of glucose clearance upon i.p. administration of glucose or insulin (Figure 4D and 4E respectively) at 6 months (data not shown). Furthermore, analysis of insulin signaling in the Alb Cre + mice revealed no differences in the activation status of the insulin-regulated proteins PKB, GSK-3 and GS. Indeed, the level of insulin-induced phosphorylation of PKB and GSK-3 was similar in liver and muscle tissues of both Alb Cre + and Alb Cre – mice (figure 5A and 5B respectively). Assay of GSK-3 activity from Alb Cre + liver extracts revealed a 50% reduction in kinase activity compared to Alb Cre – control animals (figure 5C). In addition, the extent of insulin- and glucose-induced dephosphorylation and activation of GS in muscle and liver tissues was comparable in Alb Cre + and Alb Cre – mice (Figures 6A and 6B) and the amount of glycogen deposited within in these tissues was also unaffected by the loss of hepatic GSK-3α (Figures 6C and 6D).

**Figure 7:**
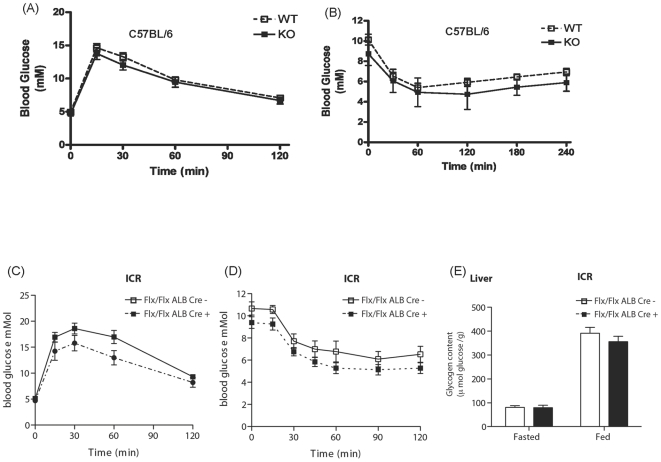
Glucose tolerance and insulin sensitivity in C57BL/6-GSK-3α global and ICR-GSK-3α liver KO animals. Blood glucose concentration in eight week old male WT - (open squares) and C57BL/6-GSK-3α KO (filled squares) mice was measured at the indicated times following administration of (A) 2 mg/g glucose or (B) 1 mU/g insulin by i.p. injection as described in Experimental Procedures. Values are the mean ± SEM from at least seven separate animals. Blood glucose concentration in eight week old male ICR-GSK-3α Alb Cre − (open squares) and ICR-GSK-3α Alb Cre + (filled squares) littermate control mice was measured at the indicated times following administration of (C) 2 mg/g glucose or (D) 1 mU/g insulin by i.p. injection as described in Experimental Procedures. Values are the mean ± SEM from nine separate animals for Alb Cre − and ten separate animals for Alb Cre +. Glycogen content was measured in liver (E) from ICR-GSK-3α Alb Cre − (open squares) and ICR-GSK-3α Alb Cre + (filled squares) following either an overnight fast or random feeding. Values are mean ± SEM from at least five separate animals with each assayed in triplicate.

Since there was a lack of insulin-sensitization in the tissue-specific deletions of GSK-3α on the C57BL/6/129 background, we next questioned whether a “true bred” C57BL/6-GSK-3α global KO mouse could reproduce the improved metabolic phenotype observed on the previously reported ICR background [11]. Interestingly, unlike GSK-3α KO mice on the ICR genetic background, we observed that animals with a global deletion of GSK-3α on a C57BL/6 background did not exhibit improved rates of glucose clearance upon i.p administration of glucose or insulin (Figure 7A and 7B). This result indicated that there might be strain-specific effects of GSK-3 deletion in glucose metabolism. Therefore, to further dissect the mechanism by which the ICR-GSK-3α KO provoked enhanced insulin sensitivity and hepatic glucose metabolism, tissue-specific deletions of GSK-3α were generated on the ICR background. To achieve this, the C57BL/6/129 GSK-3α floxed mice were backcrossed for 5 generations onto the ICR background. Since the ICR-GSK-3α global KO mouse exhibited enhanced glycogen accumulation in the liver [11], we backcrossed the C57BL/6/129 GSK-3α Alb Cre + tissue deletor strain onto the ICR background. Similar to C57BL/6/129 global knockout of GSK-3α, the ICR-GSK-3α Alb Cre + animals did not display any significant differences in the rate of glucose clearance upon i.p administration of glucose or insulin compared to the ICR-GSK-3α Alb Cre - littermate controls (Figure 7C and 7D respectively). Furthermore, there was no significant difference detected in the amount of glycogen deposited in liver (Figure 7E) or muscle (data not shown) under fasted or fed conditions.

## Discussion

Previous data have demonstrated a beneficial role of acute GSK-3 inhibition in the treatment of diabetes and obesity. Treatment of diabetic human skeletal muscle cultures with GSK-3 inhibitors stimulates glycogen synthase activity and potentiates insulin-stimulated glucose transport and glycogen accumulation [3]. Moreover, administration of these compounds to diabetic rodents improves glucose tolerance by increasing hepatic glycogen synthesis and coordinately reducing glucose output by inhibiting gluconeogenesis [19]. Whilst GSK-3 inhibitors are unable to discriminate between GSK-3α and GSK-3β, we and others have shown that there are indeed isoform- and tissue-specific roles for GSK-3 in the regulation of glucose metabolism. Selective loss of GSK-3β in skeletal muscle improves glucose tolerance and insulin signaling, while removal of this isoform in β-islet cells can reduce hyperglycaemia in diabetic IRS-2 KO mice [12], [13]. Furthermore, genetic ablation of GSK-3α results in improved whole-body glucose tolerance that is accompanied with enhanced hepatic insulin signaling and elevated glycogen accumulation [11]. These observations led us to investigate which tissues are responsible for the phenotype observed in the GSK-3α global KO mouse. In the present study we found that mice harbouring specific deletion of GSK-3α in skeletal muscle or liver displayed normal sensitivity to insulin and normal insulin signaling. This unexpected result led us to initially infer that removal of hepatic GSK-3α is insufficient to reproduce the whole body improvement of glucose metabolism that is observed with the global GSK-3α KO mouse. However, we noted that the characterization of the global GSK-3α KO had been performed on the out-bred strain, ICR but our tissue-specific knockout models were on the C57BL/6 in-bred strain (with some 129 strain content from the deletor strains). To test for possible effects of genetic background, we back-crossed the global GSK-3α KO onto the C57BL/6/129 background. Unlike the ICR strain, we observed that global inactivation of GSK-3α on the C57BL/6/129 background did not result in improved glucose/insulin tolerance or enhanced hepatic glycogen accumulation (Figure 7) or insulin sensitivity (data not shown). This result indicated strain-specific effects of genetic ablation of GSK-3α on glucose metabolism and led us to generate tissue-specific deletions of GSK-3α on the ICR background. We speculated that hepatic deletion of GSK-3α on the ICR background would reproduce the liver-specific phenotype of the ICR-GSK-3α global KO. However, this was not found to be the case, as the ICR-GSK-3α Alb Cre + mice displayed relatively normal insulin sensitivity. These experiments demonstrate that the role of GSK-3α on glucose metabolism is complex and is sensitive to genetic background (through as yet unidentified modifiers) and depends on tissues other than, or in addition to, skeletal muscle and liver. There are numerous examples demonstrating that background strain as well as environmental factors can affect the metabolic phenotypes seen in transgenic mouse models. For instance, mice that lack IRS-2 exhibit mild to severe diabetes dependent on the background strain, whereas mice lacking the muscle glycogen subunit of protein phosphatase 1 (G_M_) can result in obesity and insulin resistance in a 129/Ola donor strain, but remain lean and glucose tolerant in a 129/SvJ background. In the present case, we find that GSK-3α-induced improvement of whole-body glucose tolerance and hepatic insulin sensitivity is apparent only within the out-bred ICR strain and not within the in-bred C57BL/6 strain. It has been reported that compared to the C57BL/6, the ICR strain is more susceptible to experimental forms of insulin resistance [20]. We speculate that the beneficial effect of GSK-3α inhibition is associated with the genetic/environmental susceptibility of insulin resistance within these rodent strains. Given the beneficial effects of GSK-3 inhibitors in obese and insulin resistant rodents [4], [6], [7], it would be interesting to determine the effects of GSK-3α KO on these models.

The current study inducates that the skeletal muscle and liver tissues are unlikely to be primary contributors to the major metabolic phenotype observed within the global GSK-3α KO mice on the ICR background. The tissues responsible for the improved glucose tolerance and enhanced hepatic insulin signaling are currently unknown. There is accumulating evidence in rodents that neuronal insulin signaling plays a significant role in the maintenance of whole–body glucose homeostasis and also that it can impinge on metabolic responsiveness to insulin in peripheral tissues [21]. Indeed, it has been shown that neuronal and pancreatic deletion of IRS-2 can independently, and co-operatively, function to display many of the metabolic phenotypes observed in the global IRS-2 null mice [22], [23], [24]. Interestingly, there are reports suggesting that hypothalamic insulin signaling contributes to the regulation of hepatic glucose production [25], [26], [27]. Intra-cerebral ventricular (ICV) infusion of insulin results in the inhibition of glucose production as well as of the expression of gluconeogenic genes in the liver, independently of circulating insulin and other glucoregulatory hormones [26]. Thus, based on these findings and considering the lack of effect of hepatic deletion of GSK-3α reported here, we postulate that enhanced neuronal insulin signaling may contribute to the improved metabolic and liver-specific phenotype observed in the global GSK-3α KO on the ICR background. Indeed, we previously reported that these mice are leaner and exhibit reduced levels of circulating leptin [11]. Since neuronal pathways also influence these parameters, this provides some support for our hypothesis for a role of GSK-3α within the brain that governs whole-body metabolism and impinges on hepatic function. Indeed, we are currently exploring the functional impact of neuronal GSK-3α in the global KOs by examining insulin and leptin signaling within specific brain regions (e.g. hypothalamus).

In summary, we demonstrate that genetic background plays an important role in modulating the effect of inactivation of GSK-3α on insulin responsiveness and that mice harbouring tissue-specific inactivation of GSK-3α in skeletal muscle and liver tissues display normal insulin sensitivity. These findings contrast with the strain-specific improvement of glucose and insulin sensitivity that occurs in GSK-3α KO mice on the ICR genetic background, indicating that other, unknown tissues contribute towards this phenotype.
